# Exploration of Plant-Microbe Interactions for Sustainable Agriculture in CRISPR Era

**DOI:** 10.3390/microorganisms7080269

**Published:** 2019-08-17

**Authors:** Rahul Mahadev Shelake, Dibyajyoti Pramanik, Jae-Yean Kim

**Affiliations:** 1 Division of Applied Life Science (BK21 Plus Program), Plant Molecular Biology and Biotechnology Research Center, Gyeongsang National University, Jinju 660-701, Korea; 2 Division of Life Science (CK1 Program), Gyeongsang National University, Jinju 660-701, Korea

**Keywords:** plant microbiome, genome editing, CRISPR/Cas, plant disease resistance, plant growth promotion

## Abstract

Plants and microbes are co-evolved and interact with each other in nature. Plant-associated microbes, often referred to as plant microbiota, are an integral part of plant life. Depending on the health effects on hosts, plant–microbe (PM) interactions are either beneficial or harmful. The role of microbiota in plant growth promotion (PGP) and protection against various stresses is well known. Recently, our knowledge of community composition of plant microbiome and significant driving factors have significantly improved. So, the use of plant microbiome is a reliable approach for a next green revolution and to meet the global food demand in sustainable and eco-friendly agriculture. An application of the multifaceted PM interactions needs the use of novel tools to know critical genetic and molecular aspects. Recently discovered clustered regularly interspaced short palindromic repeats (CRISPR)/Cas-mediated genome editing (GE) tools are of great interest to explore PM interactions. A systematic understanding of the PM interactions will enable the application of GE tools to enhance the capacity of microbes or plants for agronomic trait improvement. This review focuses on applying GE techniques in plants or associated microbiota for discovering the fundamentals of the PM interactions, disease resistance, PGP activity, and future implications in agriculture.

## 1. Introduction

In nature, plants and animals continuously interact with numerous microbial species during all the stages of the life cycle. From early times of evolution, humans are exposed to a rich microbial world that extends the human capacity to adapt to a healthy life [1]. Similarly, plants cohabit with microbes, including archaea, protists, bacteria, and fungi, together called microbiota [2]. The beginning of microbial life dated back to the beginning of life (more than 3.5 billion years), suggesting that microbe–microbe interactions have evolved and diversified over time, long before the adaptation of plants to the land life, i.e., before 450 million years [3]. Higher plants and photosynthetic algae assimilated cyanobacterial endosymbionts in the process of evolution, now we know them as chloroplasts or plastids [4]. Thus, the evolutionary history of plant and microbes share common origins, and their survival is interdependent. Consequently, the “plant microbiota” has gained more attention that exists within or nearby surfaces of the plant parts [5].

Profiling of the plant-associated microbiome (genome assemblies of all microbes) is an emerging concept to understand the plant–microbe (PM) interactions. Microbiota extends the plant capacity to acclimatize fluctuating environmental conditions through several mechanisms. Beneficial PM interactions include plant growth promotion (PGP), protection against biotic and abiotic stresses through the priming of plant immune system or induction of plant defence pathways, adaptation to a variable environment, mycorrhizal symbiosis, nutrient uptake, and conversion of the unavailable nutrient forms into plant-accessible form (summarized in Reference [3]). The PM interactions are bidirectional, and microbes also obtain nutrients from the host plants. The trade-off between plant and microbe may develop into distinguishing partnerships depending on its impact on plant health, i.e., mutualistic (beneficial to both the partners, symbiotic), neutral (beneficial to only one partner, commensalistic), or harmful (deleterious to the host plant, pathogenic) [6,7]. These PM interactions are crucial in sustainable agriculture and the environment for food production and health management, respectively.

An investigation of the host plant together with associated microbiome (also called holobiont) suggests the coevolution of plant–microbe, plant–plant, and microbe–microbe interactions [8]. Modern technologies such as next-generation sequencing (NGS), omics approaches (metagenomics, transcriptomics, proteomics, metabolomics), and computational tools enable the understanding of community-level molecular aspects of the PM interactions governing the plant traits. Recently, several reports investigated the diverse aspects of plant microbiota and the influence of host genotype on different facets of the microbiome (Table 1).

Genetic information about the PM interactions is becoming available for several crops and associated microbes. Understanding the molecular aspects of PM interactions at gene level will be a crucial step toward the better use of microbiome in agriculture [14,32]. In this regard, revolutionary techniques such as CRISPR (clustered regularly interspaced short palindromic repeats)-based genome editing (GE) capable of inducing precise genetic modifications [33], are an ideal platform to know the basics of the PM interactions in a fast-forward way and enable precise genetic modifications for higher crop productivity and disease resistance [34]. In this review, we summarize a critical assessment of recent updates about the PM interactions regarding composition, structure, and factors shaping the formation of plant microbiota. We further discuss the CRISPR-based tools and their applications in the beneficial (symbiotic) or harmful (pathogenic) PM interactions towards the development of sustainable agricultural practices. We also elaborate the limitations, regulatory issues, and possible future paths while applying CRISPR-mediated techniques for agricultural purposes in plants or microbes.

## 2. Composition and Driving Factors of the Plant–Microbe (PM) Interactions

### 2.1. Composition

**Figure 1:**
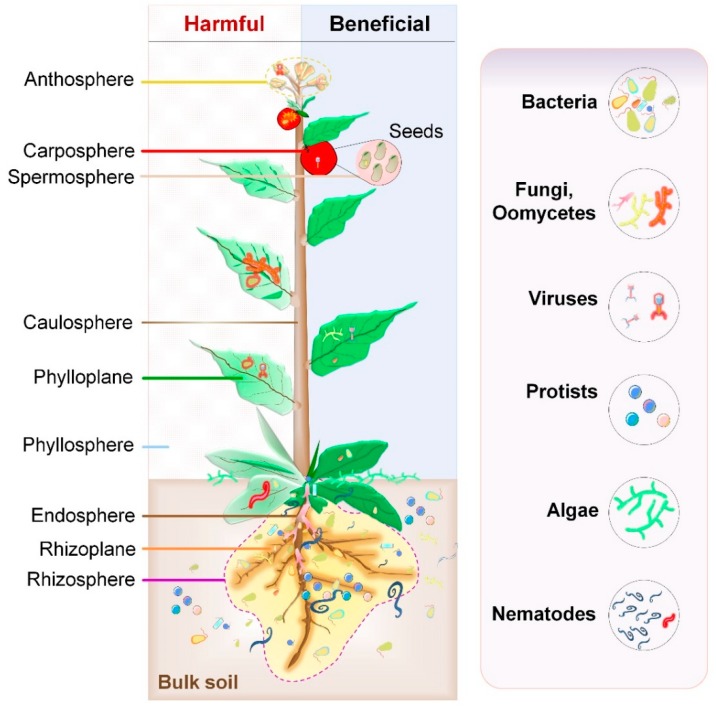
Microbiome in plant ecosystem. Schematic plant and plant-associated microbiota colonizing different niches on and inside the plant tissue. All the above-ground plant parts together, called the phyllosphere, are a continuously evolving habitat due to ultraviolet (UV) radiation and altering climatic conditions. It is primarily composed of leaves. Below-ground plant parts, mainly roots, are generally influenced by soil properties. Harmful interactions affect the plant growth through pathogenic activities of some microbiota members (left side). On the other hand, beneficial microbial interactions promote plant growth (right side).

Below-ground microbial habitat consists of the rhizosphere (soil close to the root surface), rhizoplane (root surface), and endosphere (root interior). On the other and, above-ground habitat (phyllosphere) of microbes comprises of leaves (phylloplane), stem (caulosphere), flowers (anthosphere), seeds (spermosphere), and fruits (carposphere) (Figure 1). Plants actively recruit microbes from the environment through the soil or air [35]. Some microbes such as endophytes (dwell inside the plant tissues without causing any harm to host plants) follow either horizontal (acquiring from the environment with each new generation) or vertical (transfer from parental seeds) routes of transmission [36]. The NGS-mediated profiling of microbial composition residing below- and above-ground habitats of several plants have provided thorough details about community structure, including agave [9], *Arabidopsis* [10,11,12,13,21], wild and cultivated barley [14], citrus [15], grapevine [16,17], maize [18,19,20], petunia [21], potato [22], rice [23,24], soybean [25], sugar beet [26], sugarcane [27], tomato [28,29], wheat [25,30], cucumber [30], wild mustard [31], and lettuce [37].

Although several metagenomic studies imply the abundance of the bacterial population in microbiota composition, other microbes such as viruses, protozoa, fungi, oomycetes, nematodes, and algae are also vital contributors (Table 1). Due to the use of diverse sampling methods, primers, and sequencing techniques, it is not possible to compare data from these studies [38]. Nonetheless, all of them suggest that the composition of bacterial communities contain only a few dominant phyla (Actinobacteria, Proteobacteria, Firmicutes, and Bacteroidetes). Several microbial species were found to be common between leaf- and root-associated microbiomes in *A. thaliana*, agave, grapevine, and wild mustard [9,11,14,17]. The composition of leaf microbiome, but not root, is genetically controlled by host plants and several bacterial species of leaf microbiomes are shared with root microbiomes, suggesting their acquisition from soil [31]. Overall, several reports imply that the structure of microbiota is context-dependent and consists of conserved microbial taxa dwelling in a given plant part across multiple host species and environments. However, more studies are needed to understand the effect of driving factors on microbe–microbe, plant–plant, and PM interactions during plant growth and development.

### 2.2. Factors Influencing Microbial Communities and PM Interactions

**Figure 2:**
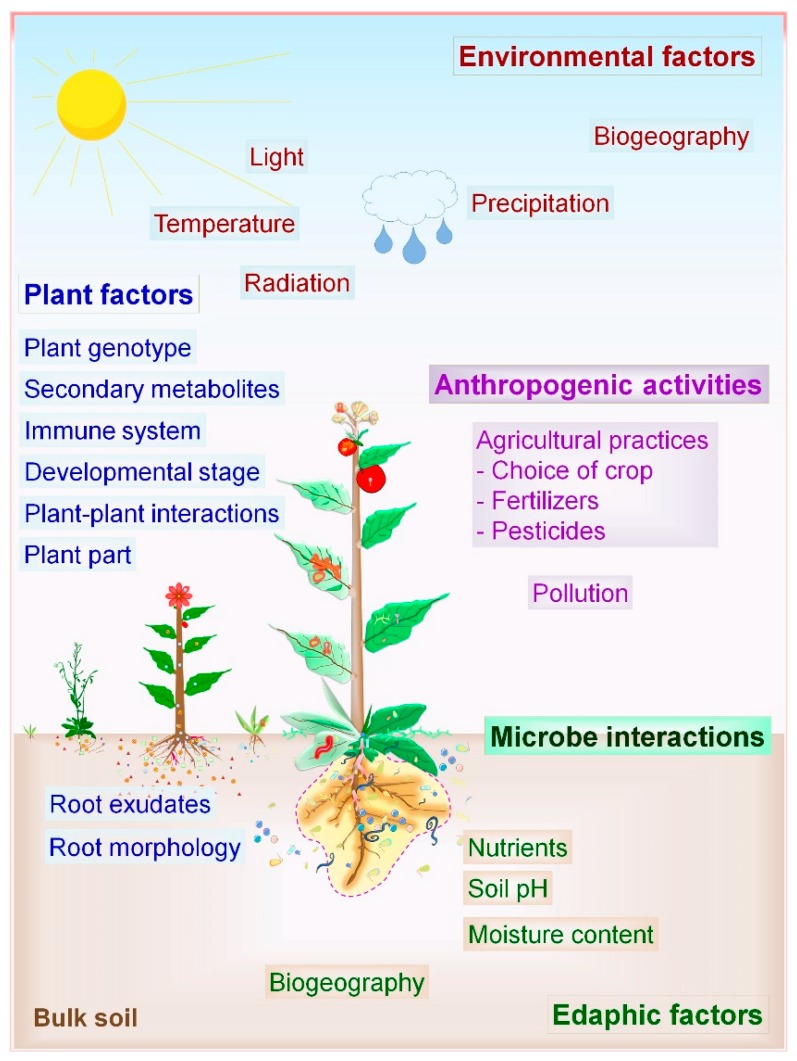
Driving factors of plant–microbe interactions. Environment-, soil- and plant-mediated factors determine the composition and structure of host microbiota. Furthermore, plant–plant, microbe–microbe, and plant–microbe interactions also impact the plant and soil microbiome.

The microbial composition of a plant microbiota is not the result of random selection, and instead, it determined by assembly rules [3,38,39]. Microbial diversity decreases sequentially from bulk soil to rhizosphere. Plant-associated factors promote the preferable growth of a set of microorganisms and inhibit the other. Recent advancement in the mapping of plant microbiota with NGS provides deep insights about biotic (plant-related factors, microbial factors, anthropogenic activities) and abiotic factors (soil properties, environmental factors) affecting the composition and structure of the microbial community (Figure 2) [3,20,38,40]. The profound effects of various factors governing the PM associations have been thoroughly reported in the model plant *Arabidopsis*, trees, and crops (Table 1).

#### 2.2.1. Biotic Factors

Plant factors include host genotype, the immune system, plant compartment, metabolite secretions, plant age, plant–plant interactions, root morphology, and root exudates. Among them, plant genotype is the major driving factor governing the composition and structure of the microbial community in the rhizosphere [41]. Different plant species vary in their rhizosphere and phyllosphere communities. It is not surprising given that plant genotype decides the properties of root and leaf surface, the type of exudates secreted by roots, chemical signalling pathways, and nutrient quantity plus quality available for microbes [14]. Plant metabolites such as coumarins affect the host microbiota, the assembly of root microbiome, and act as semiochemicals in PM interactions [42]. Microbial species available at specific geographical locations per se also influence the composition of the microbiota and their interactions with plants. The joint action of the plant–plant, microbe–microbe, and PM interactions determines the distinct microbiome assemblies [14]. Symbiotic microbial species are abundant in the niche of rhizosphere and phyllosphere due to positive and selective plant pressure. On the other hand, plant-pathogenic microbes cause a dramatic shift in the population of antagonistic microbes as well as plant immune responses, leading to the control of pathogen [13,43]. 

Anthropogenic factors such as agricultural practices, including higher dosages of fertilizers, pesticide sprays, cultivation practices pollution, and several other human activities disturb the quality of soil, air, and water, thereby influencing microbial structures and PM associations [12,21,23]. Regarding fertilizer use, the composition of root microbiota of petunia and *Arabidopsis* varied significantly in response to the phosphorus (P) application and plant species responded differently to low-phosphate conditions [21]. Furthermore, similar effects were observed for leaf microbiota in maize and soybean in response to nitrogen (N) dosages [44,45]. In the future, it will be exciting to study the impact of intensive agricultural practices on changes in PM associations and structure of plant microbiota.

#### 2.2.2. Abiotic Factors

Soil properties have a profound impact on the composition of bacterial and fungal communities in the rhizosphere [22]. The soil is the natural resource of nutrients and hence acts as a microbial seed bank for the rhizosphere community. Soil properties such as soil pH, soil type, macronutrient distribution, soil organic matter, salinity, soil structure, and moisture content drive the microbial community formation [24,25]. Plant species recruit distinct microbial communities in both rhizosphere, rhizoplane (epiphytes, colonize plant surfaces), and endosphere (endophyte, colonize internal plant parts) even if grown in a similar soil environment. On the other hand, certain plant species or genotype recruit the matching group of microorganisms irrespective of environmental and soil conditions, known as the core plant microbiota [46]. Environmental factors also significantly influence the assemblies of phyllosphere microbes that include climate, light, water, ultraviolet (UV) radiation, and geographic location [10,16,18,30]. Generally, plant phenotype is the outcome of interactions between plant genotype, associated microbiota, and environmental factors. Overall, plant microbiota is vertically (through seed, propagation material) or horizontally (through soil, air) acquired and resides on or in the inside of plant tissues where all of the above-discussed factors shape the structure of the microbial community.

## 3. Role of Plant Microbiota in Sustainable Agriculture

### 3.1. Beneficial PM Interactions in Agriculture

Plant–microbe interactions regulate the process of soil carbon sequestration by the modulating of the terrestrial carbon cycle [47]. The plant microbiota includes beneficial, neutral, or pathogenic microbial species that decompose the plant residues and dead animals. The beneficial plant microbiota is vital for plant growth, flowering time, and crop yields directly or indirectly [41,48,49]. Furthermore, microbial responses drive the impact of climate changes on agriculture, so there is growing interest to use plant-associated microbiota to mitigate the influence of climate change on sustainable agricultural practices and food production [32]. The consequence of a specific PM interaction is reliant on its effect on plant health, and it may be beneficial under the distinct set of conditions and damaging under the others. Well-known examples of beneficial PM associations include symbiotic interactions of legumes with N-fixing rhizobia and arbuscular mycorrhizal (AM) fungal taxa that helps host plants to access N and P respectively, under nutrient-deficit environments [50,51]. Symbiotic behavior helps PGP microbes to dominate the population of other microbial species. Many PGP bacteria affect plant growth via the production of phytohormones (auxin, cytokinin, gibberellin) and plant-beneficial enzymes (1-aminocyclopropane-1-carboxylate deaminase). Some PM interactions are beneficial under heavy-metal stress through enhanced uptake, and detoxification by either or both the partners, i.e., plant or microbe [52].

### 3.2. Harmful PM Interactions

Some microbes are harmful to plants causing disease symptoms, for example, *Pseudomonas syringae*, *Erwinia amylovora*, *Ralstonia solanacearum*, *Xanthomonas* sp., and *Xylella fastidiosa*. Plant-pathogenic microbes infect the plant tissues through natural openings or wounds for nutrient acquisition and trigger the immune responses [53]. Various factors regulate the outcome of plant–pathogen interaction like population size, the host vulnerability, the climate, and biotic factors like plant microbiota [54]. Several members of plant microbiome known to enhance plant resistance against phytopathogenic microbes are called biocontrol agents. Some non-pathogenic microbes can act as pathogens under some circumstances such as change of host plants [35] or alteration in microbial population size. Therefore, modern tools could be an ideal platform to understand such mysterious behavior of PM associations. In general, precise information of PM interactions at the molecular level is needed.

## 4. Modern Tools to Explore PM Interactions

Understanding the basic mechanisms of the plant-specific microbiome is a suitable approach for its use in agriculture since the plant-associated microbiota greatly influences the host’s phenotype, as described above. More precisely, the investigation of microbial and plant genes involved in the PM interactions is vital for the future application of plant microbiome. Molecular biology, omics tools (genomics, transcriptomics, proteomics, metabolomics), and NGS technologies have significantly improved our understanding of plant microbiome that includes the PGP microbes as well as phytopathogens [55,56,57,58]. For example, a shift in the composition of the beneficial microbiota community reported in plant–pathogenic interactions [19,29,59]. Although some metagenomic and proteogenomic reports provided assembly level data of augmented functional categories, alternate methods are required to gain functional insights at the gene or protein level.

**Figure 3:**
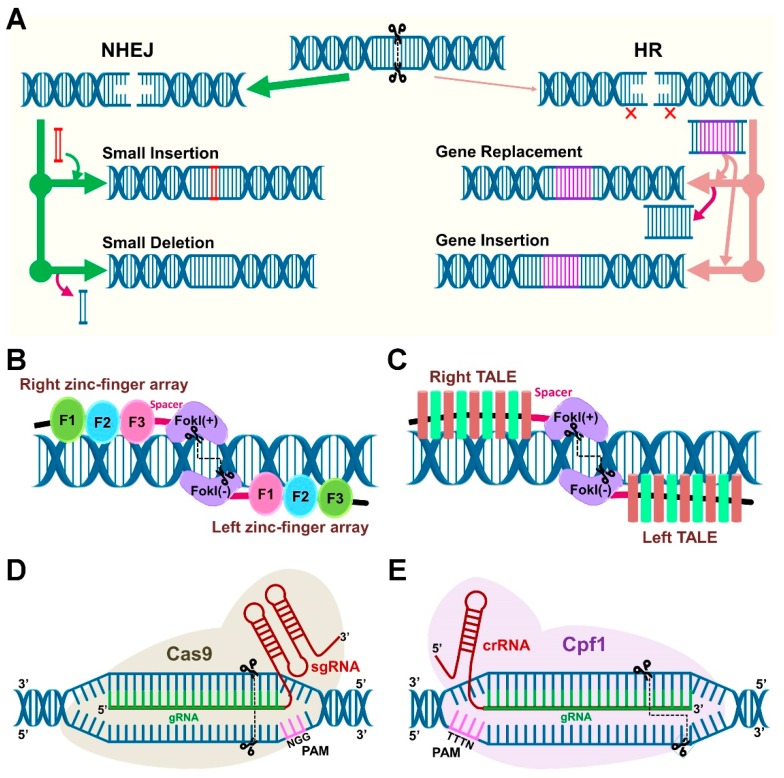
Biology and components of site-specific nucleases (SSNs) modified for genome editing applications. (**A)** Repair of double-strand breaks (DSBs) in damaged DNA strands occurs through two main pathways that consist of non-homologous end-joining (NHEJ) and homology-directed repair (HDR). NHEJ is most common in cells. It is an error-prone pathway that introduces indel mutations (small insertions or deletions). HDR is more precise compared to NHEJ, but it requires a donor template that results in either insertion or replacement. (**B**) Zinc finger nuclease (ZNF) is designed using an array of DNA-binding domains from zinc-finger proteins. Each ZFN comprises DNA-binding domain at N-terminus and FokI nuclease at C-terminus. The linker and spacers are shown in black and pink, respectively. (**C**) Design of transcription activator-like effectors (TALE) protein-based nuclease (TALEN) bound to DNA. (**D**) Illustration of clustered regularly interspaced short palindromic repeats (CRISPR)/Cas9 with a sgRNA (red) encoding gRNA (green) bound to a target DNA (blue) adjacent to PAM, i.e., protospacer adjacent motif (magenta). Cleavage sites on both strands shown with scissors and dotted line depict the blunt ends produced by Cas9. (**E**) CRISPR/Cpf1 (Cas12a) system shown with a crRNA (red), a gRNA (green), target DNA (blue), PAM (magenta). Cleavage sites of both strands (scissors) produce staggered ends (dotted line) with Cpf1.

One of the research themes about PM application in agriculture deals with the use of microbial consortia (a group of species) whereas another research theme involves a precise genetic modification (GM) of either plant or microbe. In the past, the GM methods, together with gene silencing, are widely used to study gene functions or trait improvement. Transgenic technology is a promising approach to accomplish a faster outcome, but the integration of foreign genetic material limits its widespread use due to regulatory issues [60]. In this regard, GE tools are of great interest that allows scientists to edit genomic sequences in a more precise manner without the integration of a foreign gene [33]. Genome editing technology employs the engineered endonucleases to create a double-strand break (DSB) that undergo DNA repair by endogenous mechanisms and generate different types of mutations [61]. DSB repair mechanisms occur through two major pathways (Figure 3A), non-homologous end-joining (NHEJ) and homology-directed repair (HDR). HDR is precise but less common than NHEJ, and applicable in specific donor-dependent gene replacement. 

Targeted genetic modifications can be accomplished through several ways, but three meganucleases (or site-specific nucleases (SSNs) or site-directed nucleases (SDN)), are the most commonly used recently that consist of transcription activator-like effector nucleases (TALENs), zinc finger nucleases (ZFNs), and CRISPR/Cas (Cas, CRISPR-associated) system, [62]. Genome editing by ZNFs and TALENs is based on the ability of DNA-binding domains that can specifically recognize almost any target DNA sequence (Figure 3B,C). Therefore, the GE ability of ZNF/TALEN is mainly governed by the DNA-binding affinity and specificity of the assembled zinc-finger and TALE proteins [61]. The CRISPR/Cas system is adventitious compared to ZFNs and TALENs in terms of simple designing, versatility, cost-effective, higher efficiency, multiplexing, and specificity [62]. Since the adaptation of CRISPR/Cas systems for GE in eukaryotes [63,64,65,66], it has emerged as the most popular GE tool that is also described as nature’s toolbox and ‘magic wand’ of genome engineering [67,68]. Thus, primarily CRISPR-mediated GE tools and their applications in PM studies are discussed in the following sections.

## 5. Components of the Clustered Regularly Interspaced Short Palindromic Repeats (CRISPR)/Cas System

The CRISPR/Cas is a Type II bacterial immune system found in several prokaryotes including bacteria and archaea. Ishino et al. [69] were the first to report some components of CRISPR in *Escherichia coli*, but the function of these components was not known at that time. A decade ago, the function and mode of action of CRISPR arrays as a programmed immune prokaryotic system against phages was characterized [70,71]. Soon, the components of the CRISPR immune system were reprogrammed for CRISPR-mediated GE. The two main components of CRISPR-based tool include the single gRNA (sgRNA) and Cas endonuclease [63]. The chimeric sgRNA include guide RNA (crRNA) and trans-activating RNA (tracrRNA) (Figure 3D). The key factor for using CRISPR machinery is the presence of protospacer adjacent motif (PAM), a short recognition sequence adjacent to gRNA. The sgRNA designing for the chosen target gene is simple. Several companies offer services for sgRNA synthesis, and plasmid constructs (Cas endonuclease) are available at Addgene repository (https://www.addgene.org/) that make CRISPR technology available for scientists with affordable prices. Also, multiplexing with CRISPR tools is a convenient way to perform editing at multiple targeted loci in a single cell [64]. Cas9 from *Streptococcus pyogenes* is the first Cas enzyme harnessed for GE, and it is also the most commonly used for development and application of CRISPR-based tools because of robust efficiency and PAM availability [33].

### 5.1. Cas9 and Cpf1 Orthologs

The gRNA-driven-Cas9 endonuclease scan the target genome for its complementary sequence along with PAM, after recognition, Cas endonuclease produces DSBs [66]. By designing the gRNA next to PAM, this synthetic CRISPR/Cas system can be programmed to target theoretically any desired DNA sequence for genetic modifications. Apart from Cas9, many Cas9 orthologs with the diverse PAM recognition sequences have been discovered. Some of the examples include SpCas9 from *Streptococcus pyogenes* (5′-NGG-3′) [63], StCas9 from *Streptococcus thermophiles* (5′-NNAGAAW-3′) [72,73], NmCas9 from *Neisseria meningitidis* (5′-NNNNGMTT-3′) [74], SaCas9 from *Staphylococcus aureus* (5′-NNNRRT-3′) [75], FnCas9 from *Francisella novivida* (5′-NGG-3′) [76], CjCas9 of *Campylobacter jejuni* (5′-NNNVRYAC-3′) [77], ScCas9 from *Streptococcus canis* (5′-NNG-3′) [78], and CasX (5′-TTCN-3′) [79], whereas N is any nucleotide, R is A/G, M is A/C, and W is A/T, V is G/C/A, R is A/G, Y is C/T. Among them, native spCas9 is widely used in plants [80]. Another popular Cas9 ortholog is Cpf1. Unlike Cas9, the trans-activating RNA (tracrRNA) is not required for pre-crRNA processing in Cpf1 [81]. Also, Cas9 cleavage produces blunt ends, whereas Cpf1 cutting generate staggered ends (Figure 3D,E). The reprogrammed Cpf1 orthologs for GE include FnCpf1 from *Francisella tularensis* subsp. *novicida U112* (5′-TTV/TTTV/KYTV-3′) [81], LbCpf1 from *Lachnospiraceae bacterium* ND2006 (5′-TTTV-3′) [81], AsCpf1 from *Acidaminococcus* sp. BV3L6 (5′-TTTV-3′) [81], and MbCpf1 from *Moraxella bovoculi* 237 (5′-TTV/TTTV-3′) [82].

### 5.2. Cas9 and Cpf1 Variants

**Figure 4:**
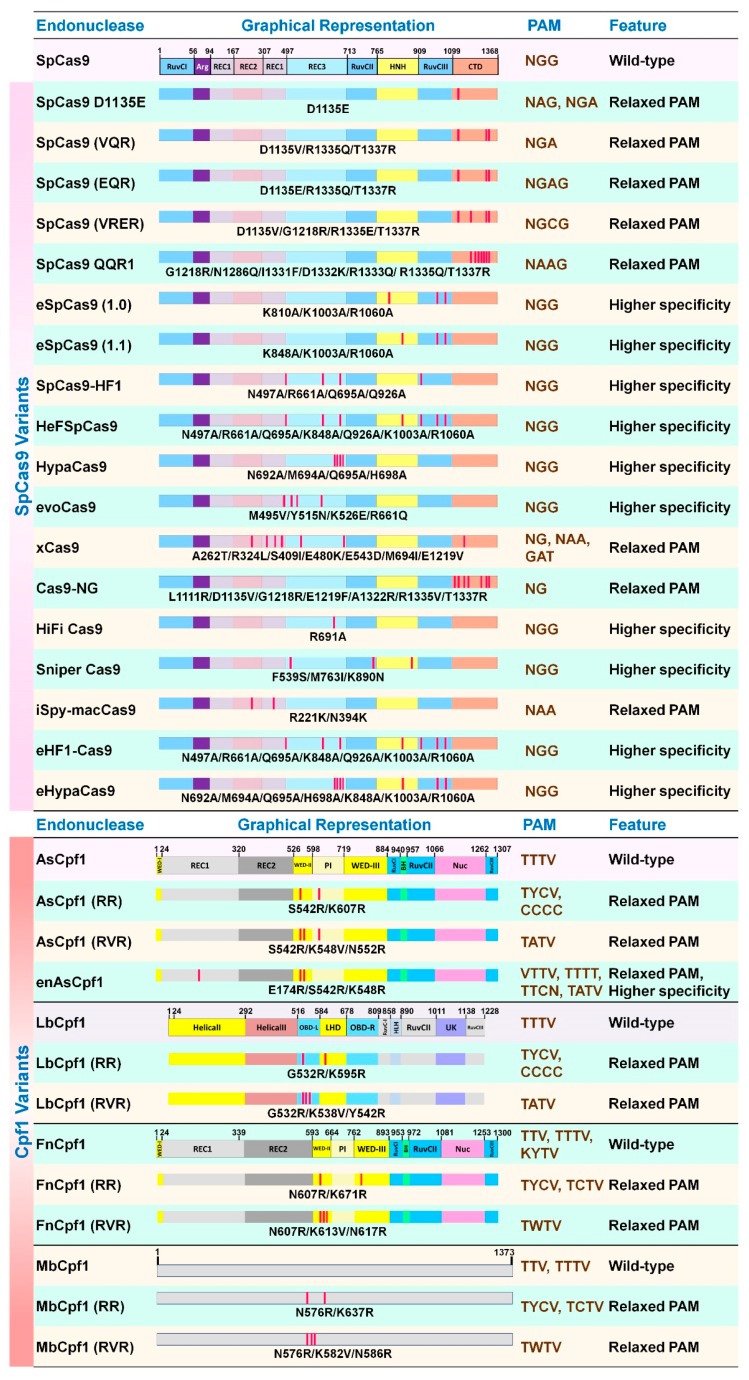
Schematic representation of Cas9 and Cpf1 variants including domain organization. Color scheme and nomenclature to represent domains followed from published structures for each enzyme. MbCpf1 structure is not yet available, hence protein domains are not drawn. Abbreviations: RuvCI-III, RuvC nuclease domain; Arg, arginine-rich bridge helix; REC1-3, recognition lobe; HNH, HNH-like nuclease domain; CTD, C-terminal domain; WEDI-III, wedge domain; PI, PAM-interacting domain; BH, bridge helix; Nuc, novel nuclease domain; OBD, oligonucleotide-binding domain; LHD, looped-out helical domain; HLH, helix-loop-helix; UK, domain with unknown functions.

Two mutant versions of Cas9, catalytically inactive cas9 (dCas9, D10A, and H840A double mutant) [63,78] and nickase Cas9 (nCas9, either D10A or H840A single mutant) [83], have been manipulated for diverse applications. For instance, base editors fused with dCas9 or nCas9 have been developed for specific C-to-T [84] or A-to-G [85] mutations in DNA. Besides, SpCas9 and Cpf1 variants have been engineered either for higher specificity or variable PAM recognition (Figure 4). Some examples of the SpCas9 variants include: SpCas9(D1135E) [64], SpCas9(VQR) [86], SpCas9(EQR) [86], SpCas9(VRER) [86], SpCas9(QQR) [87], eSpCas9(1.0) [88], eSpCas9(1.1) [88], SpCas9-HF1 [89], HeFSpCas9 [90], HypaCas9 [91], evoCas9 [92] xCas9 [93], Cas9-NG [94], HiFiCas9 [95], Sniper Cas9 [96], iSpmacCas9 [95], eHF1-Cas9 [97] and eHypaCas9 [97]. Also, some Cpf1 variants include AsCpf1(RR) [98], AsCpf1(RVR) [98], enAsCpf1 [99], LbCpf1(RR) [98], LbCpf1(RVR) [98], FnCpf1(RR) [82], FnCpf1(RVR) [82], MbCpf1(RR) [82], and MbCpf1(RVR) [82]. 

These Cas9/Cpf1 variants and orthologs provide alternatives to access different targets in the genome that are otherwise not possible to target with 5′-NGG-3′ or 5′-TTTV-3′ as PAM using native SpCas9 and Cpf1, respectively. Even though higher specificity of Cas9 may not be a major concern in plant or microbe, it is critical for clinical applications to avoid off-target effects [100].

### 5.3. RNA-Targeting Endonucleases

Some Cas9 cousins from bacterial immune systems also have been redesigned for RNA-editing, such as C2c2 or C2c6 (Cas13a from *Leptotrichia shahii* or Cas13b from *Porphyromonas Prevotella*) [101,102]. The Cas13-gRNA system has also been developed for RNA base editing, where catalytically inactive Cas13b fused to the adenosine deaminase domain of ADAR2 (Adenosine deaminases acting on RNA) for programmable A-to-Inosine (G) conversion in transcripts in human [103]. A most recent addition to CRISPR toolbox is the C-to-U RNA editor designed by directed evolution of ADAR2 into a cytidine deaminase [104]. This RNA-editing system is also applied for knockdown of RNA transcripts in plants, for example, in rice (*Oryza sativa*) protoplasts [105]. Recently, the Cas13-based molecular detection system, described as Specific High-Sensitivity Enzymatic Reporter Unlocking (SHERLOCK), was developed for pathogen identification and genotyping [106,107] as well as for detection of multiple plant genes in a single reaction [108].

## 6. CRISPR-Based Programmed Tools and Applications

**Figure 5:**
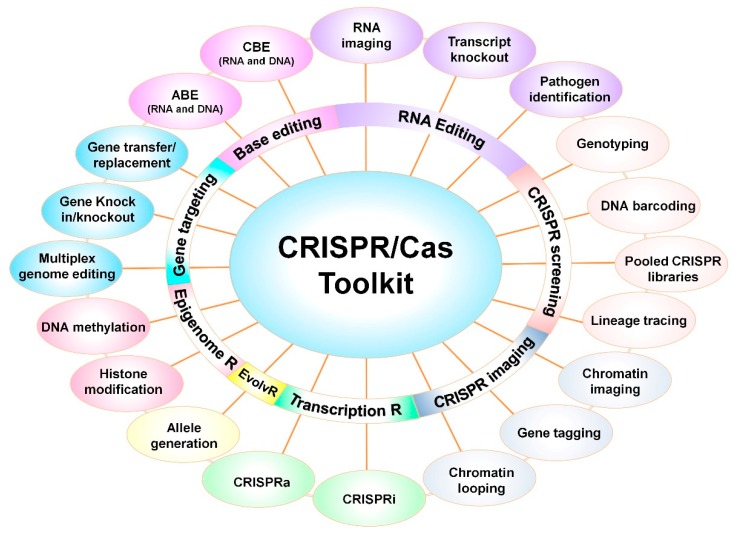
Expanding CRISPR toolkit, including applications beyond genome editing, are shown. The CRISPR-based tools have been designed for various applications in diverse fields. The SpCas9-mediated knock-out or knock-in strategy is most widely implemented in prokaryotes and eukaryotes. Catalytically dead Cas9 (dCas9) or nickase Cas9 (nCas9) and Cas9 orthologs fused with specific modulators have been reprogrammed to perform base editing (ABE, adenine base editor; CBE, cytidine base editor) RNA editing, screening libraries, chromatin imaging, transcription regulation, allele generation using the EvolvR system, and epigenome editing. Further details of each technique discussed in the main text.

Apart from the use of the CRISPR/Cas system as GE technology, programmable tools have been developed using modified Cas9 versions (i.e., nCas9 and dCas9). These CRISPR-based tools demonstrated its use in single base editing, gene regulation, epigenetic editing, chromatin engineering, imaging, and many more yet to come (Figure 5) [109,110,111]. The next generation CRISPR-based tools expanded beyond DSB-based GE and imparted the capability to these tools to precisely target the DNA region. 

The endonuclease capability of native (wild-type) SpCas9 has been explored in basic research to generate knock-out or knock-in mutants to study the gene function [63,64,65,66], and also to improve HR-based donor DNA integration at specific locus [112]. To understand the complex regulatory network, the multiplex CRISPR system is more powerful and allows targeting multiple sites with the desired manner [113]. EvolvR is a novel, targeted mutagenesis tool, designed to incorporate semi-random mutations at a dCas9/gRNA-targeted site in plasmid or prokaryotic genome [114]. The fusion of impaired Cas9 (nCas9 or dCas9) with cytidine deaminase or adenine deaminase was employed by David Liu’s group to generate cytidine base editor (CBE) and adenine base editor (ADE), respectively [84,85]. The CBE and ABE make nucleotide substitutions from C-to-T and A-to-G, which is already applied in plants [115] and microbes [116]. Additionally, mRNA trafficking was visualized in living cells by RNA-targeted Cas9 (RCas9) that could facilitate *in vivo* RNA tracking of desired transcripts [117]. 

CRISPR-based screening tools such as nucleic acid detection [106,107], gene tagging [118], barcoding [119], and lineage tracing [120], along with functional-specific genomic library [121] allows screening of the target precisely from a large population. Live-cell imaging CRISPR tools with single or multiple fluorescent proteins are highly efficient to understand the chromatin structure [122], chromatin dynamics [123,124], and topology by manipulating chromatin loops between regulatory genomic regions [125]. The CRISPR/cas9 system has been re-programmed to regulate the gene expression via modulating the transcription, translation, and epigenome.

In the CRISPR activation (CRISPRa) tool, dCas9 was fused with activator (VP64 domain) proteins to activate the transcription [66] that is also effectively applied in plants [126] and bacterial species [127], whereas the dCas9-based CRISPRi (interference) system uses the repressor complex to suppress the gene transcription [126,128]. The Cas13 has been shown to knock-out mRNA of the targeted gene, thereby suppressing the gene function [103]. Furthermore, dCas9-based epigenetic effectors are successfully applied to reactivate silenced genes or transposons through histone modification and catalysis of DNA demethylation in human [129] and plants [130]. 

Overall, pertaining to expanding the CRISPR toolbox, the CRISPR has emerged as a user-friendly GE tool for a safer and faster alternative to transgenic GM technology and time-consuming traditional breeding methods, respectively.

## 7. CRISPR-Mediated PM Applications in Agriculture

Several reports have documented the use of CRISPR-based tools in plants with particular emphasis on plant–pathogen interactions to develop disease resistance [34,80,131,132]. Here, we highlight the potential use of CRISPR technology in PM interactions to meet the food demand of the growing population sustainably through discovering the critical mechanisms of the PM interactions, developing disease resistance varieties, and enhancement of PGP activity.

### 7.1. Understanding the Fundamentals of the PM Interactions

The PM interactions are reliant on the genetics of both the microbiome and the host [133]. Some pathways of the plant immune system evolved to recognize molecules secreted by associated microbiota that serve as signals to trigger the host protection against phytopathogens [134]. Also, plant hormones such as ethylene, jasmonate, and salicylic acid, act as signals that facilitate PM interactions [135]. Especially, understanding the ‘Red Queen’ dynamics (coevolutionary cycles perpetuated due to the conflicts between pathogens and plants) of the pathogenic PM interactions will enhance our knowledge about their vital evolutionary principles (reviewed in Reference [136]).

Standard tools such as 16S ribosomal RNA-sequencing facilitate to distinguish the makeup of a microbial community. Also, multiple omics tools (gen-, transcript-, prote-, and metabol-omics) shed light on the community-level functions by plant-associated genes and pathways. However, these tools cannot determine harmful, neutral, or beneficial interaction with the host plant. Also, omics data typically emphasizes single time points of PM interactions, and thus cannot determine spatiotemporal dynamic interplays [137]. Given the significant role of microbes in plant fitness, identifying essential genes in PM interaction regulating the agronomic trait will help to improve specific plant traits for sustainable agriculture and industries. One of the critical applications of CRISPR-based GE tools is to study the gene functions by genetic modification in plants or microbes. The complete knockdown of the target gene is a unique advantage of CRISPR-based tools compared to partial gene silencing by the RNA interference (RNAi) technique that produces partial phenotype [138]. Overall, using CRISPR-based tools, we can gain more precise genetic information, particularly gene function in PM interaction at the molecular level.

Investigation of model microbes, such as root-rhizobia and phytopathogen *Pseudomonas syringae*, has provided the mechanistic understanding of genetic factors that causes mutual and pathogenic interactions with hosts, respectively [139,140]. Recently, the CRISPR/Cas system along with single-stranded DNA recombineering was established in the rhizospheric bacterium *Pseudomonas putida* KT2440 for different genetic modifications, including gene deletion, insertion, replacement, and transcription repression [141,142]. However, mechanistic studies on non-model microbial isolates are necessary to explore gene-level connections in harmful or pathogenic PM interactions. Therefore, GE of non-model microorganisms with robust CRISPR/Cas tools enable the studies to establish links between genes and functions. Further, a recent trend of using biomaterials either in DNA, mRNA, or protein form offers a unique solution for CRISPR/Cas delivery in organisms that cannot be possible by conventional methods [143]. 

### 7.2. Plant Disease Resistance

Average global yield losses due to pathogenic PM and plant–insect interactions range from 11% to 30%, mostly in the regions already suffering from food scarcity [144]. So, developing resistant crop varieties against phytopathogens has been a continuous task for agricultural scientists. Classical methods have been applied to introduce genetic modifications for improved disease-resistant plant varieties, such as crossbreeding, natural mutations, hybridization, radiation or chemical mutagenesis, and biological mutagenesis [132]. However, these methods generate several non-targeted modifications, and screening remains a time-consuming and a laborious job. In GM and GE techniques, commonly used modes for phytopathogen resistance include blocking of pathogen entry, alteration of plant defense system, modulation of recessive traits or susceptibility genes (*S*-genes), activation of dominant resistance genes (*R*-genes), expression of antimicrobial peptides, and RNAi use [145]. As discussed earlier, transgene insertion by GM technology is viewed as problematic. Also, an integrated approach (use of GM technology along with biocontrol agents, chemical fertilizers, and pesticides) to combat phytopathogens has limitations, for example, several pesticides are not efficient after pathogen evolution, and higher doses may cause harm to humans and the environment [146].

In the last decade, a new set of tools collectively labeled as new plant breeding techniques (NPBTs or NBTs), including CRISPR/Cas, have developed as an alternative (rather complementary) to classical plant breeding and GM tools [34,132]. Examples of CRISPR-mediated genetic modifications of plant or pathogen, including bacteria, fungi, oocytes, and viruses to improve disease resistance summarized in Table 2. The primary step for successful application of GE is to know the molecular details about a target gene from the plant or the pathogen. Whole-genome sequencing of several crop species and associated microbiota is available. The CRISPR-mediated disease resistance can be achieved by targeting the gene either from the host plant or pathogen.

Unlike humans and other vertebrates, plants don’t have an adaptive immune system or mobile immune cells, and are reliant on the inter-connected two-tier innate immune system to tackle pathogenic interactions [147,148]. One branch employs cell surface pattern-recognition receptors (PRRs) to distinguish between “non-self” and “self,” i.e., pathogen-associated molecular patterns (PAMPs) and plant-derived damage-associated molecular patterns (DAMPs), and initiate the PAMP-triggered immunity (PTI) [149]. The other uses resistance (R)-proteins to tackle pathogen-derived effectors to prevent the pathogen entry into the host cell and activate effector-triggered immunity (ETI) [150]. Receptor-like kinases and receptor-like proteins are known to acts as PRRs, whereas *R*-genes encode intracellular nucleotide-binding leucine-rich repeat receptor (NLRs) proteins. Such essential genes of plant innate immunity involved in the host susceptibility (*S*-genes) or resistance (*R*-genes) produced against a pathogenic response (virulence proteins or effectors) are well studied. The GE techniques targeted either of the above candidate plant genes (from PTI or ETI) or pathogen genes to confer resistance against bacteria [151,152,153,154,155,156,157,158], fungi and oocytes [159,160,161,162,163,164,165,166,167,168,169,170,171,172,173,174,175,176,177], and DNA/RNA viruses [178,179,180,181,182,183,184,185,186,187,188,189,190,191,192,193] (refer to Table 2 for details). Besides, the CRISPR/Cas system is also introduced in some microorganisms for targeted GE that act as biocontrol agents, such as insect-pathogenic fungus *Beauveria bassiana*, filamentous saprobic fungus *Purpureocillium lilacinum* controlling plant nematodes, and filamentous fungi like the *Trichoderma* species [194,195,196].

### 7.3. Plant Growth Promotion and Nutrient Uptake

Plant-beneficial free-living microbes are known as plant-growth-promoting microbes (PGPM). Among them, the most studied species include N-fixing rhizobia and arbuscular mycorrhiza (AM) fungi. For example, the plant root-associated AM fungi from phylum Glomeromycota inhabit 80%–90% of terrestrial plants and mobilize nutrients from the soil to plant [197]. Similarly, root endophytic fungus *Colletotrichum tofieldiae* isolated from *Arabidopsis* exhibits PGP activity and mobilize P to the host plant under phosphate-deficient conditions [198]. While pathogenic PM interactions have been intensely investigated to develop disease resistance in plants, little is known about several PGPM from plant microbiota involved in the beneficial PM interactions. However, the beneficial PM interactions are frequently specific to a species or a cultivar. Plant signaling mechanisms engaged in beneficial effects are very similar to the pathogenic PM interactions. But, it is not yet clear what decides the outcome of particular PM interactions [7]. The beneficial PM interaction is the result of complex processes, like enhancing the nutrient accessibility through iron uptake, N-fixation, and potassium or phosphate solubilization. These also include the activation of plant defense pathways against biotic stresses by signaling molecules, mainly systemic acquired resistance and induced systemic resistance [7]. 

Some legume root-associated bacteria and archaea have evolved the ability to convert the non-bioavailable (atmospheric) N into available (ammonium) form. This symbiotic plant-rhizobia association involves the trade of food in exchange for fixed-N. A systematic study of this symbiotic interaction about nodule organogenesis may offer ways for engineering non-legume crops to host N-fixing bacteria [199]. In this regard, GE tools are already established in some model legume species, for example, *Lotus japonicus* [200], *Medicago truncatula* [201], *Glycine max* [202] and *Vigna unguiculata* L.Walp [203]. Moreover, CRISPR protocols for some rhizospheric PGPMs also designed for genetic studies that include *Bacillus mycoides* and *B. subtilis* [204]. Overall, expanding the CRISPR toolbox could revolutionize the breeding of food legumes and non-legumes to acquire efficient N-fixing rhizobia and P-solubilizing microbes. 

Detailed molecular exploration of beneficial PM interactions with GE will facilitate their field application to non-host species, organic farms, or unfertilized soils for enhanced crop productivity. Also, the use of such GE-modified beneficial microbes in agriculture is the best alternative to agrochemicals, which is notoriously incompetent [205]. Out of field-applied micronutrients and pesticides, up to 95% and 99.9% are wasted and never reach to target [206,207], causing environmental pollution. Therefore, PGPM could be better options to decrease the cost in an eco-friendly way. The application of such genetically engineered microbial inoculants can avoid the rapid decline in introduced microbial population and subsequent benefit to the crops [208]. This also facilitates the plant microbiota-mediated remediation of contaminated soils [56].

### 7.4. Metabolic Engineering

Secondary metabolites (SMs) are generally described as natural products manufactured by an organism that is not indispensable to support life and growth [209]. The plant and associated microbes produce different SMs, mostly through some chemical pathways. While these SMs are critical in plant or microbial defense pathways, many of them have been utilized as nutrients, medicines, repellants, fragrances, flavors, and coloring agents [209]. Numerous approaches have been adopted for native or heterologous production of SMs in plants [210] and microbes [211,212]. 

Several plants microbiome studies provide an interesting insight into microbial texa supporting the activity of plant SMs production. Particularly, seed microbiome of medicinal plants such as *Salvia miltiorrhiza* [213] shows an overlapping set of bacterial and fungal genera with that of maize [214], bean [215], rice [216], and rapeseed [210]. Also, PM-associated microbes share common terpenoid metabolic pathways with the host plant, signifying their potential as a repository of SM-related genes. Furthermore, the SMs production in plants varies depending on geographical location, partly due to altered microbiota suggesting a direct link between plant metabolome and associated microbiome [217]. In this regard, the CRISPR-mediated editing of PM-associated genes involved in the SM pathway provides an innovative and attractive strategy to accomplish higher production of stable and bioactive SMs.

Recently, CRISPR-based metabolic engineering studies in either microbes or plants were performed regarding the basic understanding of SMs pathways or enhanced production of SMs [218,219]. Some studies in microbes include *Beauveria bassiana* (uridine synthesis) [196], *Trichoderma reesei* (uridine synthesis) [194], *Escherichia coli* (flavonoid synthesis) [220], *Myceliophthora* (cellulase production) [221], *Aspergillus niger* (galactaric acid production) [222], *Aspergillus oryzae* (pigment production) [223], *Shiraia bambusicola* (hypocrellin production) [224], and *Aspergillus fumigatus* (trypacidin biosynthesis) [225]. Besides, some examples of CRISPR-based metabolic engineering in plants include opium poppy (morphine biosynthesis) [226], tomato (γ-aminobutyric acid, GABA) [227], *S. miltiorrhiza* (tanshinone biosynthesis) [228], *Dendrobium* sp. (lignocellulose biosynthesis) [229], *Camelina sativa* (triacylglycerol synthesis) [230], tobacco (glycan biosynthesis) [231], and *S. miltiorrhiza* (phenolic acid) [232]. Hence, it will be exciting to use CRISPR technology for editing PM interactions to discover the novel aspects of SMs pathways and modulation of those pathways for better SM productivity.

Overall, the application of CRISPR technology holds immense potential for understanding the fundamental aspects as well as applications of PM interactions in sustainable agriculture (summarized in Table 3 along with potential CRISPR tools) in the future.

## 8. Limitations and Possible Solutions

Despite its several unprecedented advantages, the CRISPR/Cas technology still has limits while using it in different studies of PM interactions and agriculture. In general, nuclease protein size, nuclease efficiency, and PAM availability can all influence the overall CRISPR outcome, including delivery, off-target effects, and lack of viable targets [233]. As most of the CRISPR-based tools are based on SpCas9, novel Cas9 orthologs are being mined and programmed to overcome the current limitations. Some of the critical issues are discussed in the following text, along with possible solutions and future implications.

### 8.1. Culture, Species Isolation, and Transformation Protocols

Research exclusively based on culture-dependent experiments have ignored the diversity of plant microbiota. Profiling of plant microbiome by culture-independent tools has transformed our understanding of the diversity and population of microbes at the community level [2,9,12,17]. Also, culturing and isolation of individual microbes are critical steps toward developing transformation, genome sequencing, improved gene, and protein details to design CRISPR-based protocols for further study. Therefore, culture media such as abundance-based synthetic inoculants (resembling *in vivo* habitats) can be used to recover specific microbes from complex microbiota [234]. A recent innovative approach to isolate single microbial candidates includes the culturing microbiota reliant on host-based media components by adjusting various parameters (reviewed by Sarhan et al. [235]). Tissue culture protocols for major crops are either well established or being developed in recent times. Therefore, plant-based culturonomics tools might provide impetus to grow previously uncultivable plant-associated microbes and ultimately, the application of CRISPR-based tools to study PM interactions. This could also enhance the knowledge about the availability of target genes governing the desired trait in plant/microbe.

### 8.2. Delivery of CRISPR-Based Tools

One of the major hurdles in CRISPR application is CRISPR/Cas delivery in plant and microbial cells. *Agrobacterium*-mediated T-DNA transformation is the traditional delivery method being used in plants. Present delivery methods are limited to specific genotypes, species, and tissues. There is an immediate need to design novel methods for CRISPR/Cas delivery in non-model plants and microbes [236]. Non-tissue culture delivery methods will facilitate genotype-independent GE, especially using meristematic or germline plant cells. The CRISPR/Cas components can be delivered as DNA, RNA, and protein. 

One of the methods comprises the split of Cas enzyme into two inactive parts that must re-assemble into catalytically active Cas to do editing at the target DNA site [237,238]. This split-Cas9 technology is offered spatiotemporal control of Cas activity, decreased frequency of off-targets using transient methods or induced promoters, and reduced size of plasmid vectors.

Recently, the CRISPR-associated transposon-based method was reprogrammed for specific DNA insertion at target sites in bacteria [239,240]. Such pioneering techniques are a necessity for precise DNA insertion without the need for major endogenous DNA repair pathways from plant or microbe.

Lately, ribonucleoprotein complexes (RNPs) and biomaterials have emerged as promising nanotechnology approaches for the direct delivery because of their tunability, easy use, and higher efficacy that cannot be made possible by other techniques [143]. The RNPs provide better control over activity with less off-target effects compared to continuously produced CRISPR components by DNA methods. Also, novel delivery methods such as shoot meristem microinjection is a viable approach for transformation of recalcitrant plants [241].

Another problem is the delivery of several gRNAs for multiplex GE while using DNA methods. The *in vivo* production and processing of multiple gRNAs by the endogenous tRNA-processing system or a simple array of multiple gRNAs was achieved in plants and broadens the scope of genome engineering [242,243]. 

### 8.3. Transgene-Free Applications

Transgene-free GE is a great advantage of CRISPR-based technology. However, removal of the transgene is difficult from plants that are not propagated by seeds, because backcrossing is not possible. In this regard, delivery via RNP methods that do not include DNA is valuable. It will help to avoid the undesirable DNA footprints in the genome of the host. Already, Cas9 protein-gRNA as RNPs has successfully delivered and been verified to perform GE in plants [244] that could also avoid insertion of foreign DNA. Likewise, improved delivery methods based on geminiviral systems could enhance the HDR-based gene targeting in plants without any foreign DNA integration [245].

### 8.4. Off-Targets, Biosafety Laws, and Regulations

Transfer of technology from the lab-to-land is a prerequisite for successful implementation in the near future. Most of the studies are proofs of concept and need to validate similar outcomes at the field level. The great power of CRISPR/Cas technology needs to be harnessed with a greater responsibility [143]. One of the major concerns of CRISPR technology is the targeting of undesirable sites in the genome, popularly known as off-target effects [52]. These off-targets can be removed in agriculture by backcrossing the CRISPR-developed variety that allows segregation and removal of possible off-targets [91]. Also, there is a chance of gain-of-function mutations caused by potential off-targets leading to higher crop productivity. In some cases, the off-target effect may be a useful tool for mutagenesis of paralogous and homeobox genes using the same set of gRNAs. Moreover, designing highly efficient and specific gRNAs or modified gRNAs could produce a lower level of off-targets [246].

Regulatory issues of engineered plants or microbes via CRISPR are debatable in different parts of the world, primarily regulating the open-field applications of engineered plants or microbes would be a challenging task. It remains unclear whether GE products will be recognized as GM organisms (GMOs) in several countries even though SSNs introduce indels or substitutions at target sites that naturally occur in evolution process [247]. However, CRISPR-modified organisms might be recognized as GMOs in certain countries, which may need to reconsider by the regulatory authorities based on the consequence and kind of genetic modification included in plants or microbes.

## 9. Conclusions and Future Directions

Understanding the fundamentals of the PM interactions and their engineering for suitable application in sustainable agriculture is the most appropriate way to meet the food demand in the future. In the past, research about PGP microbes and microbe-mediated plant protection was performed using only a few representative species [58]. Therefore, in recent years, detailed molecular studies about microbe-mediated plant benefits have been conducted to broaden the horizon of PM engineering for agriculture. The CRISPR/Cas technology has enormous potential to help scientists to understand the basics of PM interactions and to develop ideal plant/microbes relevant for agricultural application. Consequently, studying a higher number of plant species, more in-depth sequencing analyses of the plant microbiome, and meta-transcriptomic data are supplementary to understand the community-level molecular mechanisms under field conditions. Identification of individual plant or microbial candidate genes governing agronomic traits will facilitate CRISPR-based applications in sustainable agricultural practices. Essential questions to be addressed are—which plant genes allow crops to shape the rhizosphere microbiota? What are the effects of microbes on the host plant? How do plants and microbes communicate with each other? These questions will establish the direct link between an agronomic trait and gene of the plant or microbe, allowing the design of synthetic microbial communities for higher crop productivity.

## Figures and Tables

**Table 1:** Metagenomic-based studies of plant microbiota are summarized.

Host	Sampling	Key Findings	Ref.
Agave	Rhizosphere, whole plant	Microbial composition was mainly regulated by the plant compartment, while the fungal community composition was primarily determined by the plant host biogeography.	[9]
*Arabidopsis*	Root, rhizosphere	The composition of rhizospheric microbiota was found reliant on the environment rather than host species.	[10]
*Arabidopsis*	Leaf, root	Genome drafts of 400 isolates revealed a substantial overlap of genome-encoded functional capabilities between leaf- and root-derived bacteria with few significant differences.	[11]
*Arabidopsis*	Root, rhizosphere	Explored genetic network controlling the phosphate stress response influences the structure of the root microbiome community, even under non-stress phosphate conditions.	[12]
*Arabidopsis*	Roots, rhizosphere	Bacterial microbiota is indispensable for plant survival and protection against root-filamentous fungi.	[13]
Barley	Root, rhizosphere	Rhizospheric and root microbiota affect plant growth. The interactions between microbe–microbe and plant–microbe drive distinct microbiota.	[14]
Citrus	Root, rhizosphere	The core rhizosphere microbiome comprises several potential beneficial plant microbial species and detected over-represented microbial functional traits.	[15]
Grapevine	Grape must	Environmental factors, variety, and regional origins determine the unique grapevine-associated microbiota. These factors are the key to the unique taste and wine quality.	[16]
Grapevine	Rhizosphere, whole plant	Microbial composition of soil and root is primarily influenced by plant-selective pressure, soil C:N ratio, and pH. Leaf and fruit microbiota alterations correlated with soil carbon, cultivation practices, and geography.	[17]
Maize	Roots, rhizosphere	Associated microbiota showed heritable variation in community composition of rhizosphere and significant field-specific heritable variation.	[18]
Maize	Roots, rhizosphere	Assembled a simplified and representative synthetic bacterial model community containing seven dominant strains to study the community assembly dynamics that interfered with the growth of a plant-pathogenic fungus.	[19]
Maize	Root, rhizosphere	Microbiome composition varies with plant genotype, plant age, and climate events.	[20]
Petunia, *Arabidopsis*	Root, rhizosphere	Root microbiota composition and responses vary substantially in response to the varying phosphorus (P) application.	[21]
Potato	Roots, rhizosphere	Early stages of the plant showed the cultivar-dependent composition of bacterial communities, but in the flowering and senescence stages, this was not the case. Furthermore, the population of some species flourished under different ecological conditions more than the other species.	[22]
Rice	Root, rhizosphere	Endosphere, rhizoplane, and rhizosphere consist of a diverse microbiome. Cultivation practices influence the diversity of microbiome compositions at each compartment.	[23]
Rice	Root, rhizosphere	Type of soil environment (i.e., rhizosphere versus bulk soil) is a driving factor of the structure of the microbial community than the plant age.	[24]
Soybean, Wheat	Rhizosphere, root	Soil properties such as pH and nitrate content may influence the composition of root microbiome in agricultural fields.	[25]
Sugar beet	Soil after harvesting	Identified crucial bacterial taxa and genes suppressing a fungal root pathogen and showed that plant protection depends on the rhizospheric microbial community.	[26]
Sugarcane	Rhizosphere, whole plant	Microbial communities enter primarily from native rhizospheric soil and colonize plant organs in distinct patterns.	[27]
Tomato	Rhizosphere, whole plant	Distinct microbial communities found associated with different plant organs.	[28]
Tomato	Rhizosphere, whole plant	The study explored the protection role of rhizosphere microbiota against soil-borne pathogen causing wilt disease.	[29]
Wheat, Cucumber	Roots from pots	Genus or species level differences observed between the rhizospheric microbiome from diverse plant species related to environmental factors.	[30]
Wild mustard	Leaf and root	Leaf microbiome genetically controlled by the host and several bacterial species of leaf microbiomes shared with root microbiomes, suggesting acquisition from the soil.	[31]

**Table 2:** List of genome-edited plant–pathogen interactions is summarized.

	Pathogen	Disease	Host	Target Gene *(plant or pathogen)*, Interaction	GE Tool	Ref.
				*Bacteria*		
1.	*Xanthomonas oryzae* pv. oryzae	Bacterial blight	Rice	*OsSWEET14 (plant)*; Pathogen interacts with the promoter of gene and hijacks plant sugars	TALEN	[151]
2.	*Xanthomonas oryzae* pv. oryzae	Bacterial blight	Rice	*OsSWEET14* and *OsSWEET11* (*plant*); Pathogen interacts with the promoter of gene and hijacks plant sugars	CRISPR/Cas9	[152]
3.	*Xanthomonas oryzae* pv. oryzae	Bacterial blight	Rice	*OsSWEET13 (plant)*; Pathogen hijacks sucrose from plant cells	TALEN	[153]
4.	*Pseudomonas syringae* pv. tomato, *Xanthomonas* spp., *Phytophthora capsici*	Bacterial speck, Blight, and spot	Tomato	*SlDMR6-1 (plant)*; Knock-out of *DMR6* increases salicylic acid levels that induces production of secondary metabolites and PR genes	CRISPR/Cas9	[154]
5.	*Xanthomonas citri* subsp. citri	Citrus canker	Citrus	*CsLOB1 (plant)*; Susceptibility gene induced by pathogen	CRISPR/Cas9	[155]
6.	*Xanthomonas citri* subsp. citri	Citrus canker	Citrus	*CsLOB1 (plant)*; Susceptibility gene induced by pathogen	CRISPR/Cas9	[156]
7.	*Erwinia amylovora*	Fire blight	Apple	*DIPM-1, 2* and *4 (plant)*; Directly interact with the disease-specific gene of bacterial pathogen	CRISPR/Cas9	[157]
8.	*Pseudomonas syringae pv. tomato* (*Pto*) DC3000	Bacterial speck	Tomato	*SlJAZ2 (plant)*; Directly interact with coronatine produced by bacteria that helps in leaf colonization	CRISPR/Cas9	[158]
				**Fungi and Oomycetes**		
9.	*Magnaporthe grisea*, *Burkholderia glumae*	Fungal blast, bacterial blight	Rice	*OsMPK5 (plant)*; A negative regulator of rice defense response	CRISPR/Cas9	[159]
10.	*Blumeria graminis* f. sp. tritici	Powdery mildew	Wheat	*MLO-A1, B1*, and *D1 (plant)*; Confer susceptibility to fungi	CRISPR/Cas9	[160]
11.	*Uncinula necator*	Powdery mildew	Grape	*MLO-7 (plant)*; Confer susceptibility to a fungal pathogen	CRISPR/Cas9	[157]
12.	*Ustilago maydis*	Corn smut	Maize	*bW2* and *bE1 (microbe)*; To evaluate the CRISPR system.	CRISPR/Cas9	[170]
13.	*Phytophthora tropicalis*	Black pod disease	Cacao	*Non-Expressor of Pathogenesis-Related3* (*TcNPR3*) gene *(plant)*	CRISPR/Cas9	[171]
14.	*Blumeria graminis* f. sp. tritici	Powdery mildew	Wheat	Three homologs of *TaEDR1 (plant)*; Plays a negative role in plant immunity	CRISPR/Cas9	[172]
15.	*Oidium neolycopersici*	Powdery mildew	Tomato	*SlMlo1 (plant)*; Confer susceptibility to fungi	CRISPR/Cas9	[173]
16.	*Phytophthora sojae*	Damping off	Soybean	*Avr4/6 (microbe)*; Virulence proteins enter host cells and promote host susceptibility.	CRISPR/Cas9	[174]
17.	*Magnaporthe oryzae*	Rice blast	Rice	*OsERF922 (plant)*; Negative regulator of blast fungus	CRISPR/Cas9	[175]
18.	*Leptosphaeria maculans*	Blackleg disease	Canola	Histidine kinase *(microbe)*; To study resistance mechanism against a pesticide (Iprodione)	CRISPR/Cas9	[176]
19.	*Alternaria alternata*	Black molds	Sunflower	Phosphate decarboxylase pyrG, polyketide-synthase, pksA, and 1,3,8-THN reductase, brm2 *(microbe)*; To establish a CRISPR system	CRISPR/Cas9	[177]
20.	*Magnaporthe oryzae*	Rice blast	Rice	Melanin biosynthetic polyketide synthase genes *ALB1* and *RSY1*, succinate dehydrogenase enzyme *SDI1 (microbe)*; Mutations to study the pathogenicity	CRISPR/Cas9 (RNP)	[161]
21.	*Sclerotinia sclerotiorum*	White mold	Flowers, Vegetables	Oxalate biosynthesis gene *Ssoah1 (microbe)*; Mutations to study the pathogenicity	CRISPR/Cas9	[162]
22.	*Ustilaginoidea virens*	False smut	Rice	*USTA* ustiloxin and *UvSLT2* MAP kinase *(microbe)*; To study the gene function	CRISPR/Cas9	[163]
23.	*Magnaporthe oryzae*	Rice blast	Rice	*OsSEC3A (plant)*; participate in the exocyst complex and interact with defense proteins	CRISPR/Cas9	[164]
24.	*Botrytis cinerea*	Gray mold	Grape	*WRKY52 (plant)*; Transcription factor involved in response to biotic stress	CRISPR/Cas9	[165]
25.	*Fusarium oxysporum*	Wilt	Tomato, legumes, cotton	Polyketide synthase *PKS4 (microbe)*; To study gene function	CRISPR/Cas9 (RNP)	[166]
26.	*Phytophthora capsici* and *P. sojae*	Powdery mildew, Damping-off	Vegetables, soybean	*Oxysterol binding protein-related protein 1 (microbe)*; To study resistance mechanism against a pesticide (Oxathiapiprolin)	CRISPR/Cas9	[167]
27.	*Fusarium oxysporum*	Wilt	Tomato, legumes, cotton	*FoSso1* and *FoSso2 (microbe)*; For endogenous tagging of target genes	CRISPR/Cas9	[168]
28.	*Peronophythora litchii*	Downy blight	Lychee	Pectin acetylesterase, *PAE4*, and *PAE5 (microbe)*; to study the pathogenicity	CRISPR/Cas9	[169]
				**Viruses**		
29.	BSCTV	Viral (DNA)	*Arabidopsis*	Replication origin *(microbe)*	ZNF	[178]
30.	TYLCCNV, TbCSV	Viral (DNA)	Tobacco	AC1 replication-associated (Rep) protein *(microbe)*	ZNF	[179]
31.	TYCCNV, TbCSV, TLCYnV	Viral (DNA)	Tobacco	AC1 replication-associated (Rep) protein *(microbe)*	TALE	[186]
32.	TuMV	Viral (RNA)	*Arabidopsis*	*eIF4E*/exon *(plant)*; Directly interact with viral protein and helps viral replication	CRISPR/Cas9	[187]
33.	CVYV, ZYMV, PRSV-W	Viral (RNA)	Cucumber	*eIF4E*/exon *(plant)*; Directly interact with viral protein and helps viral replication	CRISPR/Cas9	[188]
34.	RTSV	Tungro (RNA)	Rice	*eIF4G (plant)*; Directly interact with viral protein and helps viral RNA replication	CRISPR/Cas9	[189]
35.	TYLCV, BCTV, MeMV	Viral (DNA)	Tobacco	Intergenic region of origin of replication (IR), capsid protein (CP), RCRII motif of Rep protein *(microbe)*	CRISPR/Cas9	[190]
36.	BeYDV	Viral (DNA)	Tobacco	Long intergenic region (LIR), Rep protein encoding gene *(microbe)*	CRISPR/Cas9	[191]
37.	BSCTV	Viral (DNA)	*Arabidopsis*, Tobacco	IR, CP and Rep *(microbe)*	CRISPR/Cas9	[192]
38.	CBSV	Brown streak (RNA)	Cassava	*nCBP-1* & *nCBP-2*/exon *(plant)*; Directly interact with viral protein and helps viral replication	CRISPR/Cas9	[193]
39.	TMV	Viral (RNA)	*Arabidopsis*, Tobacco	*ORF1a*, *ORFCP*, 3’- UTR *(microbe)*	CRISPR/Cas9	[180]
40.	TuMV	Viral (RNA)	Tobacco	*TuMV*-GFP, Helper component proteinase silencing suppressor (HC-Pro), coat protein genes *(microbe)*	CRISPR/Cas13a	[181]
41.	WDV	Viral (DNA)	Barley	Rep, MP, LIR *(microbe)*	CRISPR/Cas9	[182]
42.	CYVV	Viral (DNA)	*Arabidopsis*	*eIF4E1* gene *(plant)*; Directly interact with viral protein and helps viral replication	Cas9- PmCDA1	[183]
43.	eBSV	Viral (DNA)	Banana	Three target sites in viral genome *(microbe)*	CRISPR/Cas9	[184]
44.	CLCuKoV, TYLCV, TYLCSV, MeMV, BCTV	Viral (DNA)	Tobacco	IR, coat protein and Rep *(microbe)*	CRISPR/Cas9	[185]

BCTV, *Beet curly top virus*; BeYDV, *Bean yellow dwarf virus*; BSCTV, *Beet severe curly top virus*; CBSV, Cassava brown streak virus; CLCuKoV, *Cotton Leaf Curl Kokhran Virus*; CRISPR/Cas9, clustered regularly interspaced palindromic repeat-CRISPR-associated protein 9; CVYV, *Cucumber vein yellowing virus*; CYVV, Clover yellow vein virus; eBSV, Endogenous Banana streak virus; eIF4E, eukaryotic translation initiation factor 4E; MeMV, *Merremia mosaic virus*; ORF, open reading frame, PmCDA1- *Petromyzon marinus* cytidine deaminase 1 base editor; PR genes, Pathogenesis-related genes; PRSV-W, *Papaya ring spot mosaic virus-W*; RTSV, Rice tungro spherical virus; SWEET, sugar will eventually be exported transporter; TALEN, transcription-activator-like effector nuclease; TbCSV, *Tobacco curly shoot virus*; TLCYnV, *Tomato leaf curl Yunnan virus*; TMV, *Tobacco mosaic virus*; TuMV, *Turnip mosaic virus*; TYLCCNV, *Tomato yellow leaf curl China virus*; TYLCSV, *Tomato yellow leaf curl Sardinia virus*; TYLCV, *Tomato yellow leaf curl virus*; UTR, untranslated terminal repeat; WDV, Wheat dwarf virus; ZNF, Zinc finger protein; ZYMV, Zucchini yellow mosaic virus.

**Table 3:** Potential applications of genome-editing in plant–microbe interaction are summarized.

Trait	Present and Future Applications	Potential CRISPR Tools
Understanding the fundamentals of the PM interactions	Identification of genes involved in PM interactions	Genotyping, DNA barcoding, lineage tracing
	Study of gene function in microbe and plant	Cas9, Cpf1 (gene knock-in/knock-out, gene replacement)
	Regulation of gene expression, promoter engineering	CRISPRa, CRISPRi (transcription regulation); DNA methylation, histone modification (epigenome editing)
	Novel allele generation	EvolvR (diversification of target genomic locus)
Plant disease resistance	Functional characterization of pathogenesis-related factors	Cas9, Cpf1 (gene knock-in/knock-out)
	Phytopathogen identification	Cas13 (RNA editing tool), RNA base editors
	Development of disease-resistant plant varieties	Cas9, Cpf1 (gene knock-in/knock-out, gene replacement), ABE/CBE (base editing)
	Pyramiding of multiple disease-resistant traits	Multiplex GE
	Pesticide resistance in crops	Cas9, Cpf1(gene knock-in/knock-out, gene replacement), ABE/CBE (base editing)
Plant growth promotion and nutrient uptake	Improvement of nutrient accessibility (biological nitrogen fixation, phosphate solubilization)	Cas9, Cpf1 (gene knock-in/knock-out, gene replacement)
	Application of nodulation in non-leguminous crops through pathway engineering	Cas9, Cpf1 (gene replacement, multiplex GE)
	Improved stress resistance by signaling molecules	Cas9, Cpf1 (gene knock-in/knock-out, gene transfer/replacement)
	Engineered microbes to reduce cost and chemical use	Cas9, Cpf1 (gene knock-in/knock-out, gene replacement, multiplex GE)
Metabolic engineering	Exploration of the novel plant metabolome pathways	Cas9, Cpf1 (gene knock-in/knock-out, gene replacement, multiplex GE)
	Secondary metabolites	Cas9, Cpf1 (gene knock-in/knock-out, gene replacement, multiplex GE)
